# Treatment differences in IDH–wildtype glioma – the impact of surgery and adjuvant therapy

**DOI:** 10.1007/s11060-025-05368-4

**Published:** 2025-12-29

**Authors:** Naureen Keric, Harald Krenzlin, Felipa Lange, Alice Dauth, Christian F. Freyschlag, Oliver Schnellg, Martin Misch, Christian von der Brelie, Jens Gempt, Aleksandrs Krigers, Arthur Wagner, Dorothee Mielke, Clemens Sommer, Marc A. Brockmann, Bernhard Meyer, Veit Rohde, Peter Vajkoczy, Jürgen Beck, Claudius Thomé, Florian Ringel

**Affiliations:** 1 Department of Neurosurgery, University Medical Center Schleswig-Holstein, Lübeck, Germany; 2 Department of Neurosurgery, University Medical Center Mainz, Mainz, Germany; 3 Department of Neurosurgery, Medical University of Innsbruck, Innsbruck, Austria; 4 Department of Neurosurgery, Medical Center University of Freiburg, Freiburg, Germany; 5 Department of Neurosurgery, Medical Center Erlangen University, Erlangen, Germany; 6 Department of Neurosurgery, Charité University Berlin, Berlin, Germany; 7 Department of Neurosurgery, University Medical Center Göttingen, Göttingen, Germany; 8 Department of Neurosurgery, Technical University Munich, Munich, Germany; 9 Department of Neurosurgery, University Medical Center Hamburg Eppendorf, Hamburg, Germany; 10 Department of Neurosurgery, University Hospital Augsburg, Augsburg, Germany; 11 Institute of Neuropathology, University Medical Center Mainz, Mainz, Germany; 12 Department of Neuroradiology, University Medical Center Mainz, Mainz, Germany; 13 Department of Neurosurgery, LMU University Hospital, Munich, Germany; 14 University of Löbeck, Ratzeburger Allee 160, 23551 Lübeck, Germany

**Keywords:** DH1/2, Astrocytoma, EOR, Survival, Adjuvant therapy, MGMT

## Abstract

**Background and objectives:**

Isocitrate dehydrogenase (IDH) wildtype (wt) astrocytomas without the microscopic features of glioblastoma have high recurrence rates and were re-classified in the presence of certain molecular features as CNS WHO grade 4 tumors in the latest WHO classification of 2021. This study examines the clinical heterogeneity within this histologically defined group and explores implications for treatment decisions, with particular focus on the role of surgical resection.

**Methods:**

Data acquisition was conducted as a multi-center retrospective analysis at 6 University Hospitals (2016-2019). Patients with IDH-wt diffuse astrocytoma without histological features of glioblastoma were enrolled. Patients presenting with IDH-wt classical glioblastoma from one institution served as controls. Primary outcome parameters were extent of resection (EOR) according to RANO 2.0 criteria, progression-free survival (PFS), and overall survival (OS).

**Results:**

160 patients with IDH-wt astrocytoma (37.5 % females) and 203 patients with IDH-wt glioblastoma (43.8 % females), were enrolled. The median age in patients with astrocytoma was younger (58.1 vs. 67.6 years; p<0.0001). Mean overall survival was significantly longer in astrocytomas (36.1 ±15.1 months) compared to glioblastomas (17.9 ±2.7 months) (p<0.0001). The extent of tumor resection is a significant factor for PFS and OS in both groups. In IDH-wt astrocytoma OS is doubled after resection of more than 50% of radiographic tumor and tripled if resection of >98% is achieved. In IDH-wt glioblastoma, resection of more than 80% of the tumor volume is needed to achieve tripled OS. MGMT methylation was not associated with longer survival in IDH-wt astrocytoma (p=.2124). While concomitant radiochemotherapy (Stupp/CeTeG) was superior to monotherapy in IDH-wt glioblastoma (p=.0094) it is non-superior to sequential therapy (radiotherapy followed by chemotherapy) in IDH-wt astrocytoma (p=.1134).

**Conclusion:**

The presented data suggests that the clinical course of IDH-wt astrocytoma, is different from IDH-wt glioblastoma with an early onset and longer survival. As concomitant radiochemotherapy is non-superior in IDH-wt astrocytoma, maximum safe resection is even more important than in classical IDH-wt glioblastoma.

**Supplementary information:**

The online version contains supplementary material available at 10.1007/s11060-025-05368-4.

## Introduction

The classification of astrocytomas WHO grade II and III according to 2007 WHO classification predominantly relied on the evaluation of histopathology and immunohistochemistry (e.g., no microvascular proliferation or necrosis) [1, 2]. In recent years, molecular markers have been shown to have significant potential as a means of classifying astrocytomas, in addition to the conventional histologic criteria [1, 2]. Therefore, the 2016 WHO classification of CNS tumors included the first molecular markers to type and grade gliomas [3]. Subsequently, the Consortium to Inform Molecular and Practical Approaches to CNS Tumor Taxonomy (C-IMPACT NOW initiative) increasingly precise categorized CNS tumors based on more specific molecular markers [4]. The presence of isocitrate dehydrogenase 1 (IDH1) and 2 (IDH2) mutations has been observed in diffuse astrocytomas (WHO grade II) and anaplastic astrocytomas (WHO grade III). These mutations have been shown to be associated with a more benign clinical course, suggesting that distinct tumor subtypes may be encompassed under a unified diagnostic categorization [2, 5]. In contrast, growing evidence indicates that the unfavorable prognosis of IDH-wildtype (wt) WHO grade II and III astrocytoma reflects the presence of previously unrecognized glioblastoma [6, 7]. Nevertheless, a subset of gliomas showed similarities in magnetic resonance imaging (MRI) criteria and histological findings consistent with WHO grade II and III astrocytomas. However, the 2018 cIMPACT-NOW Update reclassified these IDH1/2 -wt histologically grade II and III gliomas as WHO grade IV tumors when certain molecular features are present [3]. Notwithstanding the reclassification, the clinical course manifests as similar but not identical to that of classic IDH-wt glioblastomas [8]. Due to the perceived more aggressive tumor behavior closer to that of IDH-wt glioblastoma, neuro-oncologists tended to apply high-grade glioma treatment regimens [5, 9–13]. However, the randomized, open-label, phase 3 CATNON trial sheds doubt on the efficacy of either concurrent or adjuvant temozolomide in IDH1/2-wt tumors [14, 15]. This lack of informed treatment data entails doubt regarding the most suitable treatment paradigm in patients with IDH-wt astrocytoma.[16–18]

The main objective of this study was to evaluate progression-free and overall survival (PFS and OS) depending on the extent of resection (EOR) and adjuvant treatment in the largest contemporary cohort of IDH-wt astrocytoma formerly classified as WHO grade II-III. It is important to note that the present study was conducted during a transitional period in neuro-oncological diagnostics (2016–2019), when comprehensive molecular characterization according to the subsequently published WHO 2021 classification was not yet routinely implemented. Therefore, it is also an objective to explore whether this heterogenous cohort demonstrates treatment responses distinct from classical IDH-wt glioblastoma.

## Methods

### Patient samples, study design, and outcome measures

A retrospective multi-center analysis of diffuse IDH-wt astrocytomas formerly classified as WHO grade II and III included surgically treated consecutive patients from six neurosurgical university departments in Germany and Austria over a period of four years (2016–2019) were collected. Original tissue samples were not re-examined for the purpose of this study. Classic IDH-wt glioblastoma from one department were used as comparator.

### Inclusion criteria

Newly diagnosed IDH-wt astrocytomas histologically graded as WHO grade II or III and classic IDH-wt glioblastomas in patients ≥18 years at the time of diagnosis and at least one postoperative follow-up ≥ 3 months. Demographic and clinical data such as sex, age at surgery, tumor location, tumor size, the extent of tumor resection, neuropathological parameters, postoperative adjuvant treatment, follow-up duration, progression rates and survival were assessed. Postoperative follow-up was conducted via clinical investigation and evaluation of neuroimages, obtained from either magnetic resonance imaging (MRI) or, in cases where MRI was not available or contraindicated, computed tomography (CT) scans. The presence of tumor regrowth in follow-up imaging was documented as progression or recurrence, evaluated in accordance with the RANO 2.0 criteria.[19]

### Extent of resection

EOR was stratified according to definition given by the RANO resect group [20]. EOR was stratified into supramarginal- (SMR), complete- (CR), near total- (NTR), subtotal resection (STR) and biopsy. Volumetric analysis of tumor size and EOR was performed on T2-weighted images, T2-weighted fluid attenuation inversion recovery images, and T1-weighted MRI images before and after applying intravenous contrast agent using a navigation planning software (iPlan 2.1, Brainlab, München, Germany).

### Molecular analysis

Pathological diagnosis was based on 2016 WHO criteria for CNS tumor classification, and the c-IMPACT NOW Update 3 [21, 22]. Tumor marker analysis was performed using established and validated methods based on the preference of the participating center. All hospitals used methylation-specific PCR (MSP) for MGMT-promotor methylation analysis. Negative MGMT methylation levels for qMSP were below the cut-off point of 0.35. IDH-mutation status was analyzed using immune-staining. Additionally, Sanger Sequencing of genomic DNA from formalin-fixed, paraffin-embedded samples was used to analyze IDH.

### Statistics

Data analysis was performed using GraphPad Prism version 10.0.0 for Mac OS (GraphPad Software, Boston, Massachusetts USA, http://www.graphpad.com). Unpaired categorical and binary variables were analyzed in contingency tables using Fisher’s exact test. For non-normally distributed variables, continuous variables were summarized as median and range, normally distributed variables as mean ± SD and categorical variables as absolute and percentage values. For the comparison of continuous variables, the Mann–Whitney U-test was chosen because the data were predominantly not normally distributed. OS was analyzed by the Kaplan–Meier method using Gehan–Breslow–Wilcoxon test. The hazard ratio was calculated using the Mantel-Haenszel test. Finally, a stepwise backward method was used to construct a multivariate logistic regression model to analyze age, ECOG, KPS, MGMT, radio-, chemotherapy and EOR as predictors of PFS and OS. A p-value < 0.05 was considered statistically significant. Adjustment for multiple testing was not performed.

### Ethical approval

Data acquisition and analysis were performed anonymously and in accordance with the Declaration of Helsinki. The study was approved by the Ethics Committees of the Medical Association of Rhineland Palatinate, Germany (No: 2020–15140-retrospektiv). According to local laws, further consent is not necessary for retrospective analysis.

### Data availability statement

All data sets analyzed in this study are available upon reasonable request from the corresponding author.

## Results

### Demographics

A total of 160 patients with newly diagnosed IDH-wt astrocytoma, and 203 patients with IDH-wt glioblastoma were included in our study. Patients presenting with IDH-wt astrocytomas were younger (58.1±1. 1 years), compared to those with IDH-wt glioblastoma (67.6±0.8 years) (*p* < 0.0001). Sex distribution was similar in both groups (astrocytoma: 37.5% female, glioblastoma: 43.8% female; *p* = 0.24). The median ECOG score at the time of admission was 1 (range 0–4; *p* = 0.72). Methylation of the MGMT promotor was detected in 38.1% of all IDH-wt astrocytomas, and 49.0% of all IDH-wt glioblastomas (*p* = 0.0579). All tumors were IDH-wt (Table 1).

**Table 1:** Baseline demographics and histology

	IDH-wt **Astrocytoma**	**IDH-wt Glioblastoma**	**P-Values**	
Patients (n)	160	203		
Age (SE, CI)	58.1 (1.1; 56–87)	67.6 (0.8; 67–71)	< 0.0001	
Sex female (%)	60 (37.5)	89 (43.8)	0.2383	
Mean ECOG (range)	1 (0 - 4)	1 (0 - 4)	0.7203	
MGMT methylation (n, %)				
No	86 (61.9)	100 (51.0)	0.0579	
Yes	53 (38.1)	96 (49.0)		
Not available	21 (13.1)	7 (3.4)		
IDH-mutation (n, %)				
wildtype	160 (100)	203 (100)	> 0.999	
mutant	0 (0)	0 (0)		
Not available	0 (0)	0 (0)		
Progression free survival	11.54±8.4	8.22±9.5	0.4804	
Overall survival	36.1±15.1	17.9±2.7	< 0.0001	

Tumor locations in IDH-wt astrocytoma were temporal (55; 30.9%), followed by frontal (41; 23.0%), parietal (39 patients; 21.9%), insular (27 patients; 15.2%), thalamic (12 patients; 6.7%) and occipital (4 patients; 2.2%). Two or more lobes were involved in 63 patients (39.4%), both hemispheres in 11 patients (6.9%). In comparison, in IDH-wt glioblastoma two or more lobes were involved in 66 patients (32.5%, *p* = 0.186) and occurred primarily in both hemispheres in 18 patients (8.9%, *p* = 0.561).

### Survival data

**Fig. 1:**
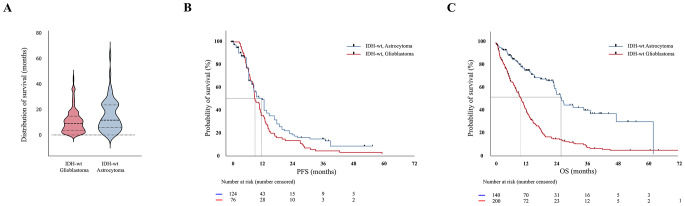
The distribution of OS in IDH-wt astrocytoma appears to be bimodal with the main peak at around 1-year and a second peak around 2-years. (**A**) Mean progression-free survival was not different between both groups. (**B**) Mean overall survival was significantly longer in IDH-wt astrocytoma (36.1±15.1 months) compared to IDH-wt glioblastoma (17.9±2.7 months) (*p* < 0.0001). (**C**)

Mean progression free survival was 11.54 ± 8.4 months in IDH-wt astrocytoma, and 8.22 ± 9.5 months in IDH-wt glioblastoma (*p* = 0.4804). The distribution of OS in IDH-wt astrocytoma appears to be bimodal with the main peak at around 1-year and a second peak around 2-years. (Figs. 1, a, b) Mean overall survival was significantly longer in IDH-wt astrocytoma (36.1±15.1 months) compared to IDH-wt glioblastoma (17.9±2.7 months) (*p* < 0.0001). (Figs. 1, 1c) Univariate analyses of predictors of PFS and OS were performed by categorizing patients according to age, sex, ECOG status, extent of resection and adjuvant treatment. Older age (IDH-wt astrocytoma: *p* = 0.0005; IDH-wt glioblastoma *p* = 0.0003) is a predictor of shorter OS in both entities. However, older patients with IDH-wt astrocytoma had a longer OS (14.1±12.7) compared to those with IDH-wt glioblastoma (9.5±1.1). (Table 2) Likewise, better functional performance (ECOG 0–2) is a predictor of better OS (IDH-wt astrocytoma: *p*= < 0.0001; IDH-wt glioblastoma: *p*= < 0.0001).

**Table 2:** Factors associated with os in astrocytoma, IDH1/2-wt (CNS WHO Grade 4) and glioblastoma, IDH1/2-wt (CNS WHO Grade 4)

	IDH-wt Astrocytoma			IDH-wt Glioblastoma			
	HR	95% CI	p Value		HR	95% CI	p Value
Age							
< 65 years	0.431	0.257–0.724	0.0015		0.598	0.442–0.809	0.0003
> 65 years	2.318	1.380–3.894			1.673	1.240–2.251	
ECOG							
0–2	0.168	0.016–1.767	< 0.0001		0.432	0.239–0.780	< 0.0001
≥3	5.930	0.566–12.74			2.317	1.282–4.188	
MGMT							
methylated	0.909	0.511–1.619	0.2124		0.694	0.512–0.939	0.0142
non-methylated	1.100	0.618–1.95			1.441	1.064–1.952	
Extent of Resection (compared to biopsy)							
SMR/CR	0.303	0.169–0.541	< 0.0001		0.277	0.168–0.456	< 0.0001
NTR	0.169	0.079–0.363	0.0026		0.313	0.194–0.506	< 0.0001
STR	0.351	0.175–0.705	0.0218		0.376	0.233–0.605	< 0.0001
PR	0.253	0.138–0.463	< 0.0001		0.740	0.449–1.220	0.253
Radiochemotherapy (compared to mono-therapy)							
Stupp/CeTeG	0.594	0.126–2.808	0.597		0.538	0.295–0.985	0.0094
Sequential therapy	0.749	0.076–7.348	0.774		-	-	-
Monotherapy	1.334	0.136–1.307	0.774		0.349	0.215–0.563	< 0.0001
No therapy	-	-	-		2.870	1.773–4.644	< 0.0001

### Extent of resection

The extent of tumor resection is a significant factor for PFS and OS in both groups. In IDH-wt astrocytoma, resection significantly prolonged PFS (mean 12.8 ± 10.6 months, HR: 0.605, 95%CI 0.333–1.06, *p* = 0.0296) and OS (mean 26.98 ± 10.6 months, HR: 0.162, 95%CI 0.088–0.2, *p* < 0.0001) compared to biopsy. Fluorescence-guided resection using 5-Ala was used in 122 patients (76.2%) with astrocytoma and 146 patients (72%) with glioblastoma. Awake craniotomies were performed more often in IDH-wt astrocytomas (32.5%) compared to IDH-wt glioblastomas (26.1%2).

**Fig. 2:**
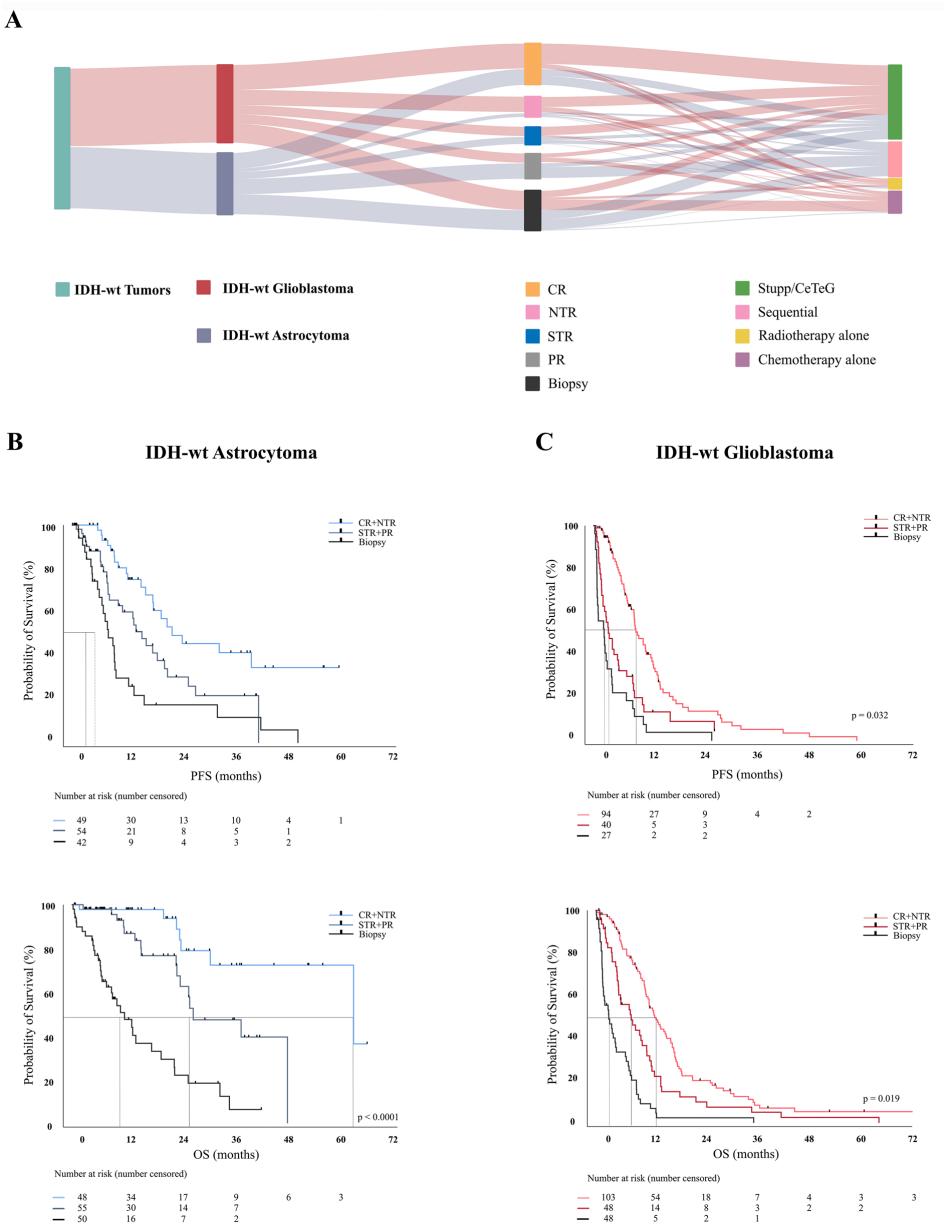
CR and concomitant therapy were used more often in IDH-wt glioblastoma compared to IDH-wt astrocytoma. (**A**) in IDH-wt astrocytoma, CR significantly prolonged PFS (*p* = 0.0031). CR, NTR and PR significantly prolonged OS (*p* < 0.005) compared with biopsy alone. (**B**) in IDH-wt glioblastoma, all types of resections significantly prolonged PFS (*p* < 0.0001) and OS (*p* < 0.0001) compared to biopsy. (**C**)

According to the RANO categories for EOR in IDH-wt glioblastoma, SMR/CR (IDH-wt astrocytoma 41 patients (25.6%); IDH-wt glioblastoma 65 patients (31.5%)), NTR (IDH-wt astrocytoma: 9 patients (5.6%); IDH-wt glioblastoma: 40 patients (19.7%)) and STR (IDH-wt astrocytoma: 40 patients (25.0%); IDH-wt glioblastoma: 24 patients (11.8%)) were achieved more often in IDH-wt glioblastoma (CR+NTR: *p* = 0.0094; STR: *p* = 0.014). No difference was found in PR (IDH-wt astrocytoma: 18 patients (11.3%); IDH-wt glioblastoma: 24 patients (11.8%), *p* = 0.99) and biopsy (IDH-wt astrocytoma: 52 patients (32.5%); IDH-wt glioblastoma: 50 patients (25.2%), *p* = 0.286). (Figs. 2, 2a) In IDH-wt astrocytoma, CR significantly prolonged PFS (*p* = 0.0031), while PR, NTR and CR all significantly prolonged OS (*p* < 0.005) compared with biopsy alone. (Figs. 2, 2b) (Table 2). Likewise, in IDH-wt glioblastoma, all types of resections significantly prolonged PFS (*p* < 0.0001) and OS (*p* < 0.0001) compared to biopsy. CR significantly prolonged PFS (*p* = 0.0326), while STR, NTR and CR all significantly prolonged OS (*p* < 0.0001, respectively) compared to biopsy alone (Figs. 2,c).

In IDH-wt astrocytoma OS is doubled after resection of more than 50% of radiographic tumor and tripled if resection of > 98% is achieved. In IDH-wt glioblastoma, resection of more than 80% of the tumor volume is needed to achieve tripled OS.

### Non-surgical treatment data

**Fig. 3:**
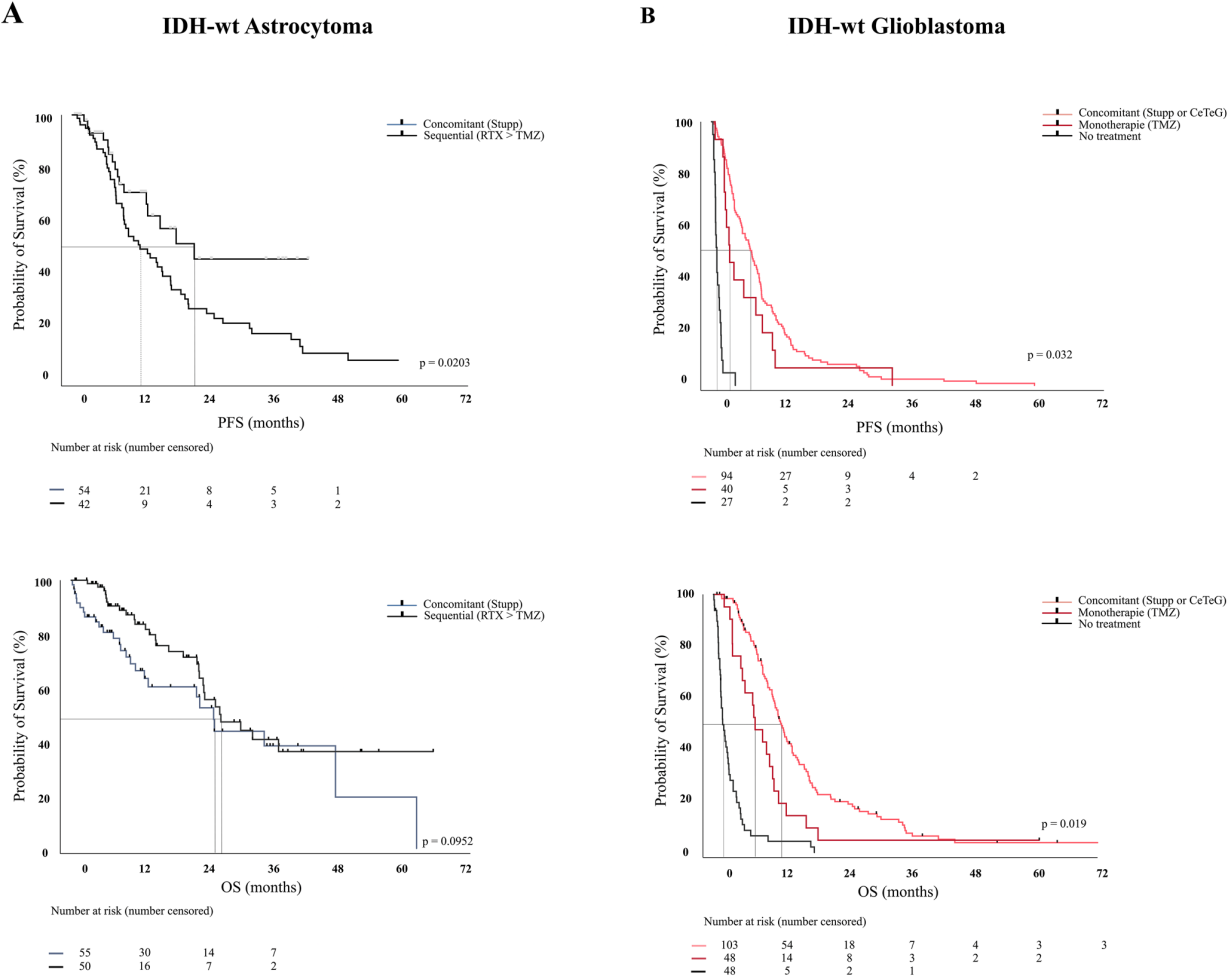
In IDH-wt astrocytoma, treatment according to stupp- or CeTeG protocol resulted in similar PFS and OS compared to those treated with radiotherapy followed by sequential chemotherapy (*p* = 0.1134). (**A**) in IDH-wt glioblastoma, concomitant radiochemotherapy (stupp/CeTeG) was superior to monotherapy (*p* = 0.0094) and each treatment regimen superior to best-supportive care (*p* < 0.0001). (**B**)

Treatment decisions were made at local interdisciplinary tumor conferences based on clinical status, the extent of resection, and histopathological findings, including molecular markers. Adjuvant treatment was performed in 151 patients (74.4%) with IDH-wt glioblastoma and 158 (98.8%) with IDH-wt astrocytoma. Many patients received concomitant treatment with temozolomide (TMZ, 75 mg/m^2^) during radiotherapy (RT), followed by 6 cycles adjuvant TMZ (150-200 mg/m^2^) for 5 days out of 28 days (Stupp protocol) (IDH-wt Astrocytoma: 56 (35.0%), IDH-wt glioblastoma: 87 (42.9%)) or concomitant treatment using TMZ (75 mg/m2) during hypofractionated (hf) RT, followed by 6 cycles adjuvant TMZ (150-200 mg/m2) for 5 days out of 28 days (Perry protocol) (IDH-wt Astrocytoma: 6 (3.8%); IDH-wt glioblastoma: 34 (16.7%)). Concomitant treatment using TMZ (150 mg/m2) and CCNU/Lomustine during hfRT, followed by 6 cycles adjuvant TMZ (150-200 mg/m2) for 5 days out of 28 days, the CeTeG protocol, (6-week courses of oral combined CCNU/TMZ (CCNU 100 mg/m^2^ on day 1, TMZ 100–200 mg/m^2^ on days 2–6) starting in the first week of radiotherapy) was considered in patients with methylated MGMT promotor in patients with IDH-wt glioblastoma only (*n* = 6 (2.9%)). Sequential treatment (radiation followed by chemotherapy) was considered in patients with former IDH-wt astrocytoma (*n* = 88, 55.0%). Other regimens included radiation as monotherapy (IDH-wt astrocytoma: 4 (2.5%); IDH-wt glioblastoma: 16 (7.9%)), Nordic radiation scheme (IDH-wt astrocytoma: 0 (-); IDH-wt glioblastoma: 4 (2.0%)) and best-supportive care. In IDH-wt astrocytoma IDH1/2-wt, treatment according to Stupp- or CeTeG protocol resulted in similar PFS and OS compared to those treated with radiotherapy followed by sequential chemotherapy (*p* = 0.1134). (Figs. 3, 3a) (Table 2) In IDH-wt glioblastoma, concomitant radiochemotherapy (Stupp/CeTeG) was superior to monotherapy (*p* = 0.0094) and each treatment regimen superior to best-supportive care (*p* < 0.0001) (Figs. 3,b) (Table 2)

.

### Molecular markers

**Fig. 4:**
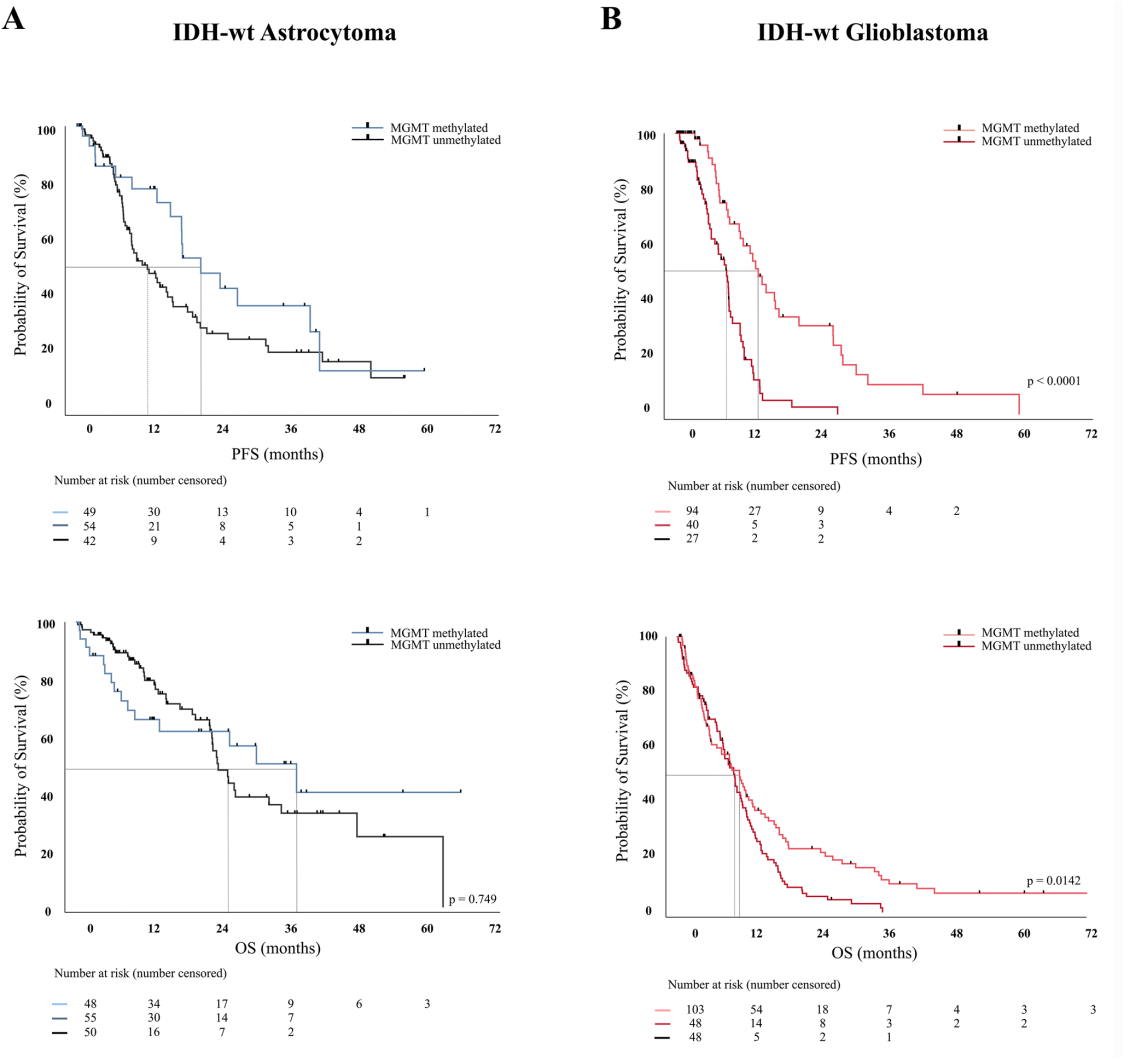
MGMT promotor methylation was not associated with longer survival in IDH-wt astrocytoma (HR: 0.909 (0.511–1.619), *p* = 0.2124) but is associated with a favorable therapy response in IDH-wt glioblastoma (HR: 0.694 (0.512–0.939), *p* = 0.0142) (**A**, **B**)

Not all molecular markers were assessed on a routine basis in the participating centers. In the cohort of the IDH-wt astrocytomas the TERT promoter was mutated in 28 of 41 analyzed patients (68.3%); EGFR amplification was detected in 23 out of 48 analyzed patients (47.9%); and nuclear ATRX loss was detected in 12 (9.6%) patients, while it was retained in 113 (90.4%). The presence of a TERT mutation and strong EGFRvIII expression was associated with an impaired survival in the respective subgroup (23.0 months, 95CI10.0–30.6; *p* = 0.011). Furthermore TERT-mutant cases were older (mean 61.8 ± 10.2 years) compared to TERT-wildtype cases (mean 54.1 ± 12.7 years), though this difference did not reach statistical significance (*p* = 0.058). MGMT-promoter methylation was examined in 140 IDH-wt astrocytoma patients; data were not available in 20 patients. In 54 patients (38.6%) the promotor was methylated and in 86 (61.4%) non-methylated (Table 1). MGMT methylation was not associated with longer survival in IDH-wt astrocytoma (HR: 0.909 (0.511–1.619), *p* = 0.2124) but is associated with a favorable therapy response in IDH-wt glioblastoma (HR: 0.694 (0.512–0.939), *p* = 0.0142) (Fig. 4) (Table 2).

### Multivariate analysis

Variables associated with statistically significant differences in survival in patients with IDH-wt astrocytoma found in univariate analyses (age > 65 years, ECOG, EOR) as well as several putatively clinically significant variables (adjuvant treatment, MGMT methylation status) were included in a multivariate survival analysis. The extent of resection SMR/CR (*p* = 0.0012), NTR (*p* = 0.0014), older age (*p* = 0.009) and unfavorable ECOG (*p* = 0.0004) remained statistically significant factors for OS. (Table 3)

**Table 3:** Association of patient- and tumor characteristics with survival (multivariate analysis) in IDH-wt astrocytomas without the microscopic features of IDH-wt glioblastoma

	HR (95% CI)	P value
**Extent of resection**		
SMR/CR	0.278 (0.125–0.594)	0.0012
NTR	0.109 (0.669–3.468)	0.0014
**Age** ( > 65 years)	2.613 (1.007–1.050)	0.009
**ECOG** (≥3)	3.57 (2.179–7.857)	0.0004
**MGMT** (methylation status)	1.156 (0.398–1.252)	0.247
**Adjuvant treatment**		
Stupp/CeTeG	0.839 (0.111–17.67)	n.s.
Sequential therapy	0.709 (0.061–9.829)	n.s.
Monotherapy	1.151(0.067–20.08)	n.s.

Multivariate Cox Proportional Hazards Regression analysis, backward conditional selection method used, step 6 is displayed for PFS and step 4 for OS

## Discussion

IDH-wt astrocytomas are a heterogeneous group of tumors that have undergone numerous diagnostic reclassifications over time. Based on molecular markers, the majority is classified as IDH-wt glioblastomas CNS WHO grade 4 according to the 2021 WHO classification.[1, 3]

Despite higher recurrence rates and poor clinical outcome, imaging features of these tumors resemble low-grade gliomas [23, 24]. These tumors lack histological criteria of classic IDH-wt glioblastoma such as microvascular proliferation and necrosis [16–18, 25]. However, higher recurrence rates and more aggressive growth patterns are observed in these tumors [5, 7, 9, 10, 26]. Increasing evidence from multiple studies suggests, that the poor clinical outcome stems from a considerable proportion of unrecognized glioblastoma in this group [5, 9]. In the presented data, distribution of survival from IDH-wt astrocytoma is distinguished into two larger groups of less and longer than 20 months. This finding confirms that different tumors are subsumed under this entity. Driven by the admixture of TERT-mutant and EGFR overexpressing cases, these tumors might cluster in a shorter survival peak. The latest WHO 2021 classification integrates the combination of histological and molecular grading by incorporating additional genetic data such as TERT promoter mutation, EGFR amplification, and/or copy number changes (7 gain/10 loss) in IDH1/2-wt astrocytomas, as outlined in the cIMPACT-NOW criteria, to define IDH-wt glioblastoma [1]. The higher proportion of TERT promotor mutations (68.3%) and EGFRvIII expression (47.9%) in those IDH-wt astrocytoma that where tested, strongly suggests that a substantial number of these cases would be reclassified as “molecular IDH-wt glioblastoma CNS WHO grade 4” according to WHO 2021 criteria. These data validate the concerns raised by the cIMPACT-NOW updates and the WHO 2021 classification regarding the heterogeneity of histologically diagnosed low-grade IDH-wt astrocytomas. The remaining 31.7% of TERT-wildtype cases of our cohort of IDH-wt astrocytomas cases may represent true IDH-wt astrocytomas without molecular glioblastoma features, molecular IDH-wt glioblastomas with alternative defining alterations (EGFR amplification or +7/-10) that were not comprehensively assessed, or a mixture of both categories. Although missing a molecular work-up of the whole cohort, these findings underscore that our cohort represents a heterogenous mixture rather than a single biological entity. This has important implications for interpretation of treatment outcomes and clinical decision-making. While prospective adjuvant treatment data remains unavailable, the subsequent treatment perception has been adapted towards the more aggressive stance used in IDH-wt glioblastoma.[16–18]

The presented data highlights the important associations between more extensive resection and outcome in IDH-wt astrocytoma in the largest contemporary multicenter cohort so far. The data provides evidence for a rather favorable clinical course compared to IDH-wt glioblastomas, even in case of comparable post-resection residual tumor volume. However, the benefit obtained by maximal safe surgical resection is substantially higher compared to classic IDH-wt glioblastoma. This observation stands in contrast to previous studies observing similar outcomes between both entities following surgery and radiation [27]. The presented data emphasize the importance of the resection of non-CE tumor portions. This observation is supported by other retrospective studies indicating that IDH-wt glioblastomas with ≤5.4 cm^3^ non-CE postoperative tumor and no residual CE tumor benefit from resection [24]. The RANO *resect* group previously published similar findings indicating that resection of non-CE tumor beyond the CE tumor margins has prognostic implications in glioblastoma arguing against conclusion from prior studies that glioblastomas with non-CE do not benefit from resection [20, 23, 28].

No difference in OS with the use of concomitant radiochemotherapy according to Stupp- or CeTeG protocol, compared to sequential therapy (radiotherapy followed by adjuvant chemotherapy) was observed in IDH-wt astrocytomas. These findings seem to align well with previous studies reporting similar survival outcomes after radiotherapy or the addition of concurrent (and/or adjuvant) TMZ [27]. It is of note, that all of the analyzed patients were treated with either concomitant or sequential radiochemotherapy. As there was no difference between both treatment strategies and none of the patients received either of these treatment modalities alone, it remains elusive if adjuvant treatment improves survival and if so, which of these treatment modalities possess the higher efficacy.

In IDH-wt glioblastoma response to alkylating chemotherapy is better in tumors with methylated MGMT promoter [29 P]romoter methylation is detected in about 40% of all patients with IDH-wt astrocytomas and IDH-wt glioblastomas [30, 31]. Similar MGMT promotor methylation rates were detected in the presented cohort. Limited data suggests a prognostic role of MGMT promoter methylation in IDH-wt astrocytomas with regard to chemotherapy response and OS [31]. The randomized, open-label, phase III CATNON trial in patients with 1p/19q non–co-deleted anaplastic gliomas indicated futility of concurrent temozolomide with radiation and adjuvant temozolomide in patients with IDH-wt tumors. Benefit was restricted to adjuvant treatment in IDH-mutant tumors [14, 15]. A post-hoc analysis from the CATNON study population, identifying 159 IDH-wt tumors with molecular features of a IDH-wt glioblastoma, similarly revealed no additional benefit of temozolomide in regard to PFS and OS compared to radiotherapy alone [32]. MGMT promoter methylation provided no clinical benefit with either concurrent or adjuvant temozolomide [14, 15]. This observation falls in line with our data revealing no survival difference given the MGMT methylation status in response to therapy and survival in IDH-wt astrocytoma as known from IDH-wt glioblastoma.

## Limitations of this study

The retrospective design is an inherent limitation of the presented study. As treatment decisions were based in local practice and judgment, it may influence outcome and survival data. Further, it was impossible to control for treatment regimens after surgery that might affect progression-free and overall survival. Because the original clinical diagnoses were included without central neuropathological review, the data was not homogenized for specific diagnostic algorithms but instead represent the clinical interpretation of current EANO and WHO diagnostic criteria of the time and therefore do not represent the most up-to-date classifications. Without systematic assessment of molecular markers, the cohort analyzed on this study likely represents a heterogenous mixture of true IDH-wt astrocytomas and unrecognized molecular IDH-wt glioblastomas. This limitation provides an opportunity to examine the clinical consequences of incomplete molecular classification and to assess whether treatment responses that might inform clinical decision-making in settings where complete molecular profiling is not immediately available. We emphasize these limitations not to diminish the clinical observations but to highlight the challenges in clinical practice during the WHO classification transition period.

## Conclusion

This study showed that the clinical course of patients with IDH-wt astrocytomas is better than that of IDH-wt glioblastoma. The presented data highlights the important associations between more extensive resection and outcome in IDH-wt astrocytoma, even in case of similar post-resection residual tumor volume. The benefit obtained by maximal safe surgical resection is substantially higher compared to classic IDH-wt glioblastoma. The data indicates the importance of non-CE tumor resection in this entity.

However, MGMT promotor methylation is of no prognostic value for survival in this cohort, while no difference between concomitant and sequential radiochemotherapy was observed. The findings of this study implicate a refined treatment paradigm starting with a maximum safe resection and followed by a tailored approach depending on the molecular characteristics.

## Electronic supplementary material

Below is the link to the electronic supplementary material.

Supplementary Material 1

## Data Availability

All data sets analyzed in this study are available upon reasonable request from the corresponding author.
